# Unraveling cooperative and competitive interactions within protein triplets in the human interactome

**DOI:** 10.1038/s41598-025-19264-4

**Published:** 2025-09-15

**Authors:** Aimilia-Christina Vagiona, Pablo Mier, Miguel A. Andrade-Navarro

**Affiliations:** 1 Insitute of Organismic and Molecular Evolution, Faculty of Biology, Johannes Gutenberg University, Biozentrum I, Hans-Dieter-Hüsch-Weg 15, 55128 Mainz, Germany; 2 Andalusian Center for Developmental Biology (CABD, UPO-CSIC-JA), Faculty of Experimental Sciences (Genetics Area), Universidad Pablo de Olavide, 41013 Seville, Spain

**Keywords:** Biophysics, Computational biology and bioinformatics, Structural biology, Systems biology

## Abstract

Knowledge of protein–protein interactions (PPIs) is essential for understanding cellular function, yet most network analyses focus on binary interactions. Higher-order motifs such as protein triplets can reveal cooperative or competitive relationships but are difficult to distinguish systematically. We present a computational framework to classify protein triplets in the human protein interaction network (hPIN) as cooperative or competitive. The hPIN was embedded in hyperbolic space using the LaBNE + HM algorithm, and a Random Forest classifier was trained on structurally validated triplets from Interactome3D, achieving high accuracy (AUC = 0.88). Angular and hyperbolic distances were key predictive features. Predicted cooperative triplets were enriched in paralogous partners, indicating that paralogs often bind together to a shared protein using non-overlapping surfaces. The model proved to be effective when tested on a new dataset. AlphaFold 3 modeling supported these predictions, showing that cooperative partners bind at distinct sites, while competitive ones overlap. Our results demonstrate the value of hyperbolic geometry for capturing functional organization in protein complexes.

**Supplementary Information:**

The online version contains supplementary material available at 10.1038/s41598-025-19264-4.

## Introduction

Proteins are the essential units of life, regulating crucial biological processes ranging from molecular transport to signal transduction^1^. These functions depend on a highly coordinated network of protein–protein interactions (PPIs), collectively referred to as the interactome, which controls cellular organization and functionality^2,3^. Understanding these interactions is key to unraveling biological mechanisms and designing therapeutic strategies for various diseases^4,5^.

Protein–protein interaction networks (PPINs) are primary constructed from binary interactions, which represent direct physical contact between two proteins^6^. Most large-scale interactome maps rely on pairwise interaction data, as these are the most accessible and well-characterized through traditional experimental approaches^7^. High-throughput techniques such as yeast two-hybrid (Y2H) assays and affinity purification coupled with mass spectrometry (AP-MS) have been essential in mapping the human interactome^8,9^. However, many biological processes extend beyond pairwise PPIs, as proteins often function in multi-protein assemblies rather than simple one-to-one interactions, in order to carry out essential tasks^10,11^. Among these multi-protein complexes, protein triplets represent a crucial class of higher-order interactions. Triplet interactions provide a framework for understanding cooperative and competitive dynamics, which influence the structural and functional stability of protein complexes^12,13^.

Cooperative interactions occur when multiple proteins work together synergistically to enhance stability or function, such as in multiprotein enzyme complexes or transcription factor binding events^14,15^. In contrast, competitive interactions arise when two proteins compete for the same binding partner, modulating signaling pathways or enzymatic activity based on cellular conditions^16^. Accordingly, proteins interacting with many partners can have either multiple interfaces or just one interface. Some of these interfaces are shared by different partners, resulting in mutually exclusive bindings; other interfaces are used by only one partner such that interactions with different partners can occur simultaneously. Thus, to judge whether two partners can interact with the common protein simultaneously, the key is to know whether they share an interaction interface^17^.

Despite their importance, higher-order interactions remain difficult to study using traditional experimental methods, which are primarily designed to detect pairwise interactions. This limitation highlights the need for computational approaches that can infer triplet interactions and predict whether they are cooperative or competitive. By integrating network properties and advanced mathematical models, such as hyperbolic embeddings, researchers can gain a more comprehensive understanding of how protein complexes form and function in the cellular environment^18^.

Several studies have demonstrated that complex networks, including the human protein–protein interaction network (hPIN) can be effectively modeled using hyperbolic geometry^19–21^. The Popularity-Similarity (PS) model provides a geometric framework in which network nodes are positioned within a two-dimensional hyperbolic space (H^2^), represented as a disk. In this model, the radial coordinate of a node captures its popularity and evolutionary age, with older, highly connected proteins positioned closer to the center, while newly emerging proteins occupy the periphery. The angular coordinate encodes functional similarity, grouping proteins involved in shared biological processes or pathways. Alanis-Lobato et al. demonstrated that embedding the hPIN in hyperbolic space provides biologically meaningful insights, with radial positioning reflecting protein conservation and seniority, and angular positioning capturing functional and spatial organization within the cell^22^. Such mapping approaches can contribute to a deeper understanding of complex human disorders^23–25^.

Motivated by the importance of higher-order protein interactions, we developed a computational framework to distinguish cooperative from competitive triplets in the human protein interaction network (hPIN). Starting from a high-confidence PPI network embedded in hyperbolic space, we identified open triangles, triplets of proteins where two interact with a shared partner but not with each other, and annotated them using structural data. Using this annotated dataset, we trained and evaluated a Random Forest algorithm to classify protein triplets as either cooperative or competitive based on topological, geometric and biological features. This classification is related to the previously defined “party” and “date” hubs, which distinguish proteins based on co-expression and temporal interaction patterns^26^. However, we adopt the terms cooperative and competitive to emphasize structural criteria, specifically, whether the two partners can bind the shared protein simultaneously at distinct interfaces (cooperative) or must do so mutually exclusively due to overlapping interfaces (competitive). To assess the predictive power of our approach, we performed structural validation using AlphaFold 3, confirming distinct spatial configurations between predicted cooperative and competitive triplets. By exploring multi-protein interactions, we provide a deeper insight into how molecular complexes are organized and operate within biological systems.

## Results and discussion

### Construction of the hPIN into the hyperbolic space and structural annotations of cooperative and competitive relationships

**Fig. 1:**
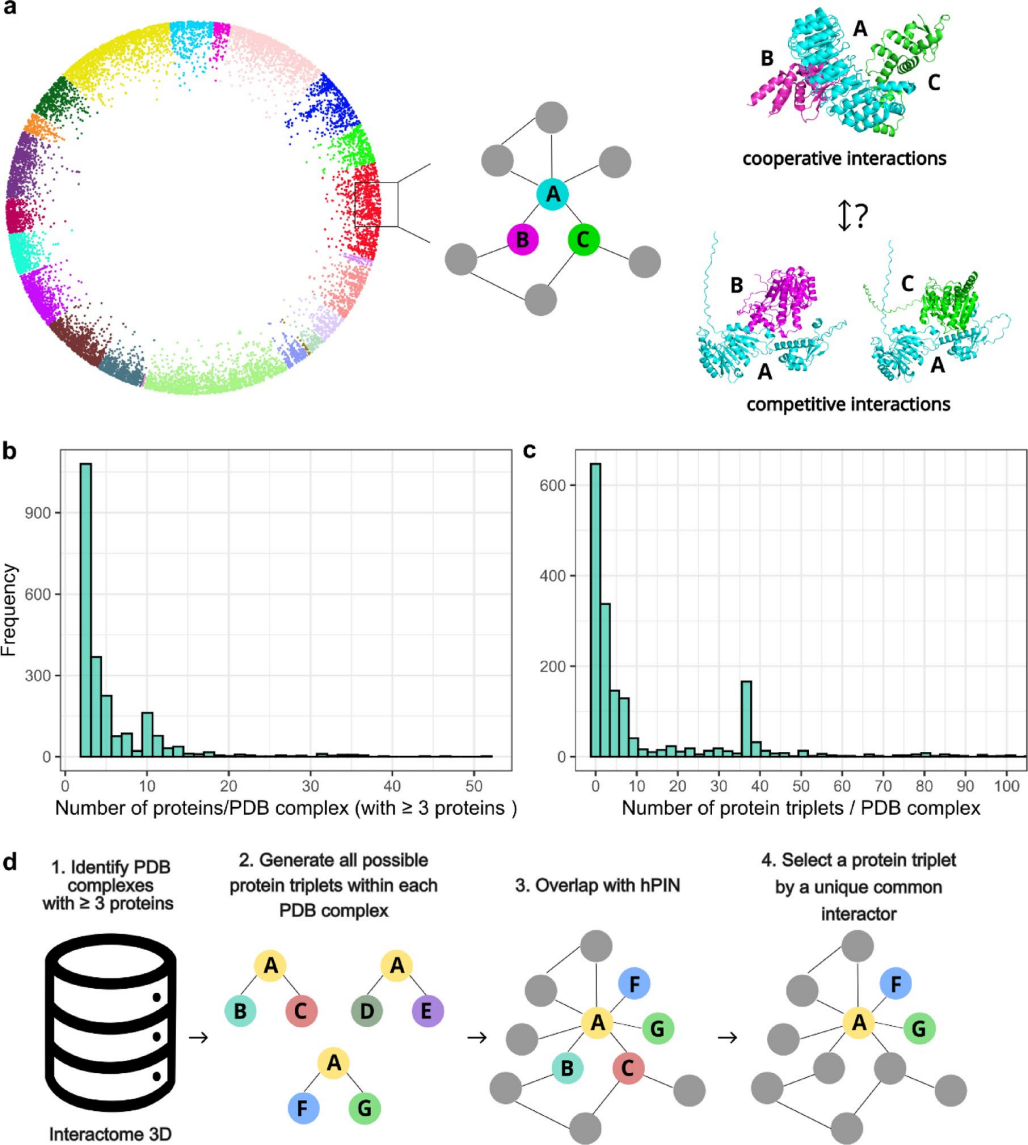
Construction of the human protein interaction network and identification of structurally supported cooperative triplets. (**a**) Overview of the high-confidence human protein–protein interaction network (hPIN) constructed from the HIPPIE database (confidence score ≥ 0.71) and embedded into two-dimensional hyperbolic space using the LaBNE + HM algorithm. Node colors represent functional clusters identified using the angular gap-based clustering method described in our previous study^18^, where proteins grouped by angular proximity reflect functional similarity. (**b**) Distribution of the number of proteins per PDB complex with at least three unique proteins, based on residue-level annotations from Interactome3D. (**c**) Distribution of the number of structurally supported open triangle motifs (i.e., cooperative triplets) per PDB complex. A subset of complexes contributes disproportionately high numbers of triplets, reflecting structurally rich assemblies. (**d**) Mapping of structurally supported triplets into the hPIN. Only one cooperative triplet was retained per common interactor to ensure non-redundancy, resulting in a curated set of 211 cooperative triplets used for model training.

To investigate cooperative and competitive interactions between proteins in triplets, where one protein interacts with another two proteins, we first constructed a high-confidence human protein–protein interaction network (hPIN) using experimentally supported data (Fig. 1a). For this, we retrieved all human PPIs from the HIPPIE database and filtered interactions with a confidence score ≥ 0.71, as this threshold ensures that the majority of the interactions are validated through multiple independent sources. The resulting network comprised 15,319 proteins and 187,791 interactions. To uncover the latent geometry underlying the hPIN, we embedded the hPIN into the two-dimensional hyperbolic plane (H^2^) using the LaBNE + HM algorithm, which integrates manifold learning with maximum likelihood estimation (see Methods for details). Each protein was assigned with a set of hyperbolic coordinates: a radial coordinate (r) representing its topological centrality where a shorter distance to the center corresponds to nodes with higher connectivity, and an angular coordinate (theta) indicating its similarity to other nodes based on interacting partners^18,22,25^. This hyperbolic embedding allowed us to extract geometric and topological features essential for the classification of cooperative versus competitive interactions within protein triplets.

To investigate the diversity of protein structures involved in cooperative triplets, we analyzed residue-level annotations from the Interactome3D database^27^. We identified triplets within experimentally resolved complexes, focusing on open triangle configurations in which a central “common” protein binds two partners (V1 and V2) that do not interact directly. Such motifs are indicative of potential cooperative relationships, especially when the binding regions of the common protein are distinct or complementary. Across all PDB complexes containing at least three proteins, we observed a broad distribution in the number of proteins per complex, with most structures comprising fewer than 15 unique proteins (Fig. 1b)^28^.

These complexes were then computationally evaluated to identify open triangles. We found that while many PDB entries contained only a few open triangles, some complexes presented high structural complexity, producing up to 100 triplets (Fig. 1c). Large well-studied assemblies such as ribosomes, proteasomes and chaperones, where many subunits are resolved together within the same structural experiment, result in large amounts of triplets^29,30^. Subunits of such large complexes participate in multiple triplets, which could be a source of bias in analyses of their properties.

After characterizing the structural diversity of complexes, we proceeded to map the identified triplets into the hPIN. This allowed us to extract structurally supported triplets with topological relevance, which served as cooperative examples for modeling. Specifically, we overlapped the common–V1 and common–V2 interaction pairs from each structurally validated triplet with the interactions present in the hPIN (Fig. 1d). To avoid the redundancy that could be introduced by counting proteins from large complexes multiple times, as mentioned above, we retained only one cooperative triplet per common interactor (see Methods for details). This filtering resulted in a final, non-redundant set of 211 cooperative triplets, distributed across 352 PDB complexes, each annotated with residue-level interface information derived from Interactome3D. These well-supported triplets formed the positive class used to train our classification model (see Data Availability section to access these data).

### Predicting cooperative triplets in the human protein interaction network

To systematically predict cooperative interactions among protein triplets, we constructed a dataset composed of positive and negative cases. The 211 structurally supported triplets identified from Interactome3D^27^ served as the positive class, representing cooperative configurations in which a central protein (common interactor) binds two partners without a direct interaction between them (V1 and V2). As a “noisy” negative class, we selected open triangles from the hPIN that lacked structural support. These contain both positives and negatives. To eliminate any potential positional bias (for example, due to labels), we randomized the assignment of V1 and V2 within each triplet.

To build our model, we extracted for each triplet a set of topological and geometric features. Topological and hyperbolic features have been previously shown to be predictive of protein interaction with function in protein (de-)phosphorylation^16^. These included hyperbolic coordinates and centrality measures (degree, closeness, betweenness, eigenvector) for each of the three proteins in a triplet, and distances, angular and radial differences for each pairwise relationship (common-V1, common-V2, V1–V2).

We also considered biological features: presence of a disordered region, and subcellular location, for each of the three proteins in a triplet. From a biological perspective, we assumed that the formation of stable cooperative complexes requires that interacting proteins are co-localized in the same subcellular compartment^31^. In addition, proteins with well-defined structural domains are often capable of forming stable binding interfaces, making structural order a key indicator for such interactions^32^.

In contrast, intrinsically disordered proteins (IDPs), or proteins with intrinsically disordered regions (IDRs), lack a well-defined tertiary structure under physiological conditions. While this structural flexibility can facilitate transient interactions such as in cellular signaling, it is generally less favorable for stable, multimeric complex formation^33^. The final feature matrix comprised 42 features per triplet (see Methods for details).

We trained multiple machine learning models—Random Forest (RF), Support Vector Machine (SVM), Logistic Regression, Decision Trees, and k-Nearest Neighbors (kNN)—and evaluated their performance using a 70/30 train-test split. Specifically, 70% of the dataset was used for model training and internal validation, while the remaining 30% was reserved for testing. To address the class imbalance between cooperative (positive) and competitive (negative) triplets, we applied random undersampling to the majority class in the training set prior to model training, resulting in a balanced dataset of 296 samples. For the training procedure, we performed fivefold cross-validation repeated 10 times.

**Fig. 2:**
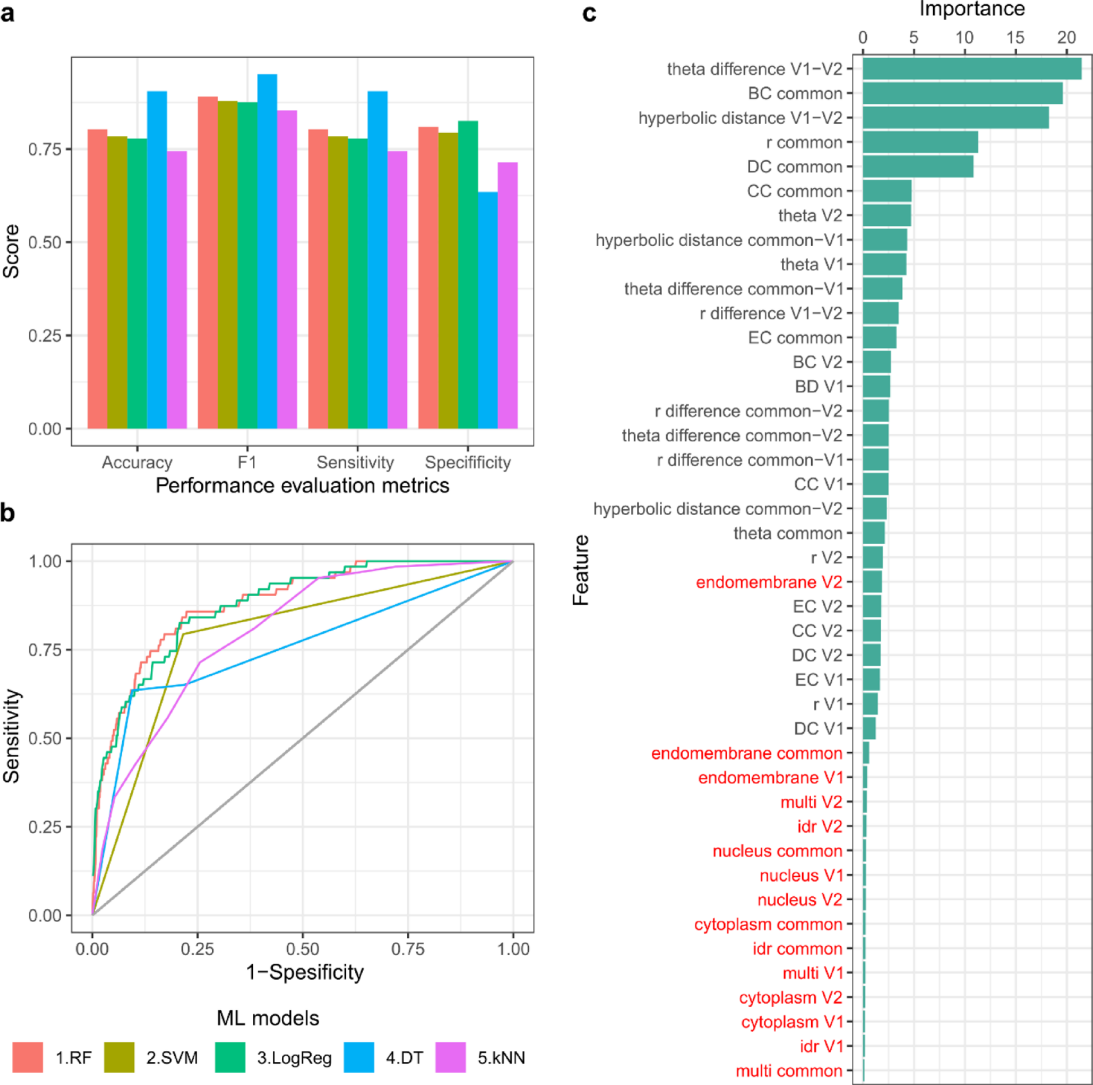
Predicting cooperative protein triplets in the human protein interaction network using machine learning. (**a**) Performance metrics (accuracy, F1-score, sensitivity, and specificity) of five machine learning models trained to classify cooperative versus competitive triplets. Random Forest (RF), Support Vector Machine (SVM), Logistic Regression (LogReg), Decision Trees (DT), and k-Nearest Neighbors (kNN). The Random Forest (RF) classifier showed the highest overall performance. (**b**) Receiver Operating Characteristic (ROC) curves for all models. The RF model achieved the best performance with an area under the curve (AUC) of 0.89, indicating strong discriminatory power. (**c**) Feature importance scores from the RF classifier, ranked by mean decrease in accuracy. The most informative features included the angular difference between V1 and V2, the betweenness centrality of the common protein, and other geometric and topological features derived from the hyperbolic embedding and network structure. Biological features (labels marked in red) were less important.

Among all tested classifiers, the Random Forest (RF) model (trained with 500 trees) achieved the highest performance, with a mean accuracy of 0.80, F1-score of 0.89, sensitivity of 0.80, and specificity of 0.80 on the held-out test set (Fig. 2a). ROC curve analysis with an AUC of 0.88 (Fig. 2b) indicated a superior ability to distinguish cooperative and competitive interactions between triplets of protein complexes. As our model predicts the interaction type within protein triplets, each unique triplet was evaluated once, resulting in a total of n = 17,165,561 triplet evaluations across the human protein interaction network. The model produces a probability score indicating whether a given triplet interaction is predicted to be cooperative or competitive. A total of 3,405,136 triplets received a score ≥ 0.5, and 311,557 triplets were classified with high confidence, receiving a score ≥ 0.9 (Supplementary Table S4).

To gain insight into which features were most predictive of cooperative interactions, we examined feature importance scores derived from the Random Forest model (Fig. 2c). The most important feature was the angular difference between V1 and V2, closely followed by the hyperbolic distance between V1 and V2 proteins. The high importance of the angular and hyperbolic geometrical features aligns with previous observations that angular positioning in hyperbolic space captures the functional organization of the proteome^22^.

Other top-ranking features included the betweenness (BC), degree (DC) and closeness (CC) centrality of the common protein as well as its radial coordinate. BC represents proteins that act like bridges in the network, DC values indicate how well a node is directly connected to the other nodes and CC measures the central position of the proteins in the network. Regarding the radial coordinate, in the hyperbolic embedding, proteins closer to the center (with lower radial values) are usually more connected in the network and often older in evolutionary terms.; they can be informative for predicting cooperation or competition between the proteins.

Together, these results highlight the value of hyperbolic embedding, which captures latent geometric and topological properties of the protein interaction network that cannot be easily inferred from biological features alone. Although biological attributes like subcellular localization and structural order add meaningful information, it was the geometric features derived from the network, especially angular and hyperbolic distances, that played the most critical role in distinguishing cooperative from competitive triplets. This demonstrates that embedding the hPIN in hyperbolic space enhances our ability to capture functional relationships and improve predictions of protein interaction types.

### Biological interpretation of predicted triplets

To investigate the biological relevance of our model’s predictions, we focused on the subset of predicted cooperative and competitive triplets where all three proteins had low degree centrality (degree ≤ 10).

This filtering step reduced the influence of highly connected hub proteins and allowed us to focus on triplets where cooperative assembly might be more functionally focused. From this subset, we retained 1595 triplets and divided them into four quantiles based on their predicted probability scores, with Q1 representing low-scoring (competitive-like) and Q4 high-scoring (cooperative-like) predictions (Supplementary Table S5).

**Fig. 3:**
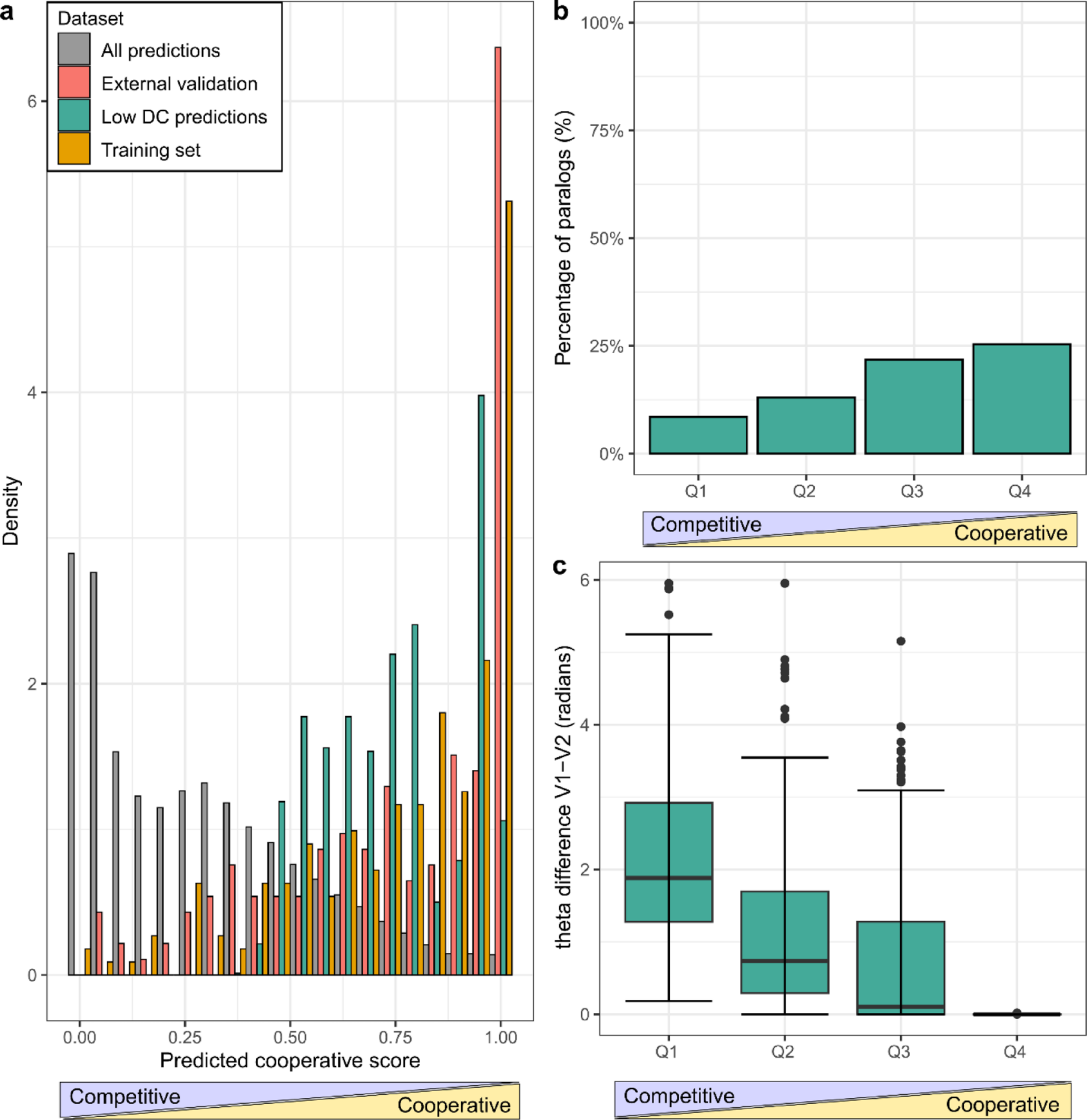
Biological interpretation of predicted triplets based on model score distributions, paralogy, and angular separation. (**a**) Distribution of predicted cooperative scores across datasets (legend). The all‑predictions set is enriched toward low scores (competitive‑like), whereas the training, external validation, and low‑degree common (DC) subsets concentrate near high scores (cooperative‑like). (**b**) Proportion of triplets in which V1 and V2 are paralogs across prediction-score quartiles (Q1 = low/competitive, Q4 = high/cooperative). Paralogous pairs are more prevalent in Q4, indicating that similar proteins are more often predicted to act cooperatively. (**c**) Distribution of angular (theta) differences between V1 and V2 across quartiles. Angular separation decreases toward Q4; cooperative triplets tend to show smaller theta differences (functional proximity), whereas competitive-like triplets display larger separations.

In addition to this subset, we evaluated the model on an external validation set derived from the most recent Interactome3D release, containing structurally supported triplets absent from the training data to ensure an independent test of predictive performance. Comparing the distribution of predicted scores across the all-predictions set, the training set, the external validation set, and the low DC subset showed distinct distributions (Fig. 3a). The all-predictions set was skewed toward lower scores (competitive), whereas the other three datasets showed a shift toward higher scores (cooperative), with the external validation subset showing the strongest enrichment. This finding confirms the model’s ability to detect cooperative assemblies in previously unseen structural data.

Next, we assessed the paralogous relationship between V1 and V2 in each triplet. Based on the idea that paralogous proteins share structural and functional similarity, we initially expected them to appear more often in competitive interactions, where they could compete for the same binding site on the common interactor. Instead, our results revealed the opposite pattern. The proportion of paralogous V1–V2 pairs was highest in Q4 and decreased gradually toward Q1 (Fig. 3b), indicating that paralog pairs are more likely to occur in cooperative triplets. This finding is consistent with previous studies showing that paralogs are frequently retained within the same protein complexes after duplication, contributing to cooperative function, structural stability, and functional redundancy rather than direct competition^34,35^.

Finally, we examined the angular (theta) difference between V1 and V2, the top-ranked feature from our model. Triplets in Q4 exhibited the smallest angular separation, with theta differences progressively increasing toward Q1 (Fig. 3c). This pattern suggests that cooperative triplets often involve proteins that are functionally similar in the hyperbolic space, consistent with paralogs or related proteins acting together within the same complex. In contrast, larger angular separations, indicative of greater functional diversity, were more frequently observed in predicted competitive triplets. These findings support the idea that cooperation is favored between proteins sharing similar functional and structural contexts, as is typical for subunits within protein complexes. This observation can be compared with the L3 principle described by Kovács et al., where proteins connected by many paths of length three tend to share interaction partners and often bind the same interface^36^.

### Structural evaluation of predictions using AlphaFold

**Fig. 4:**
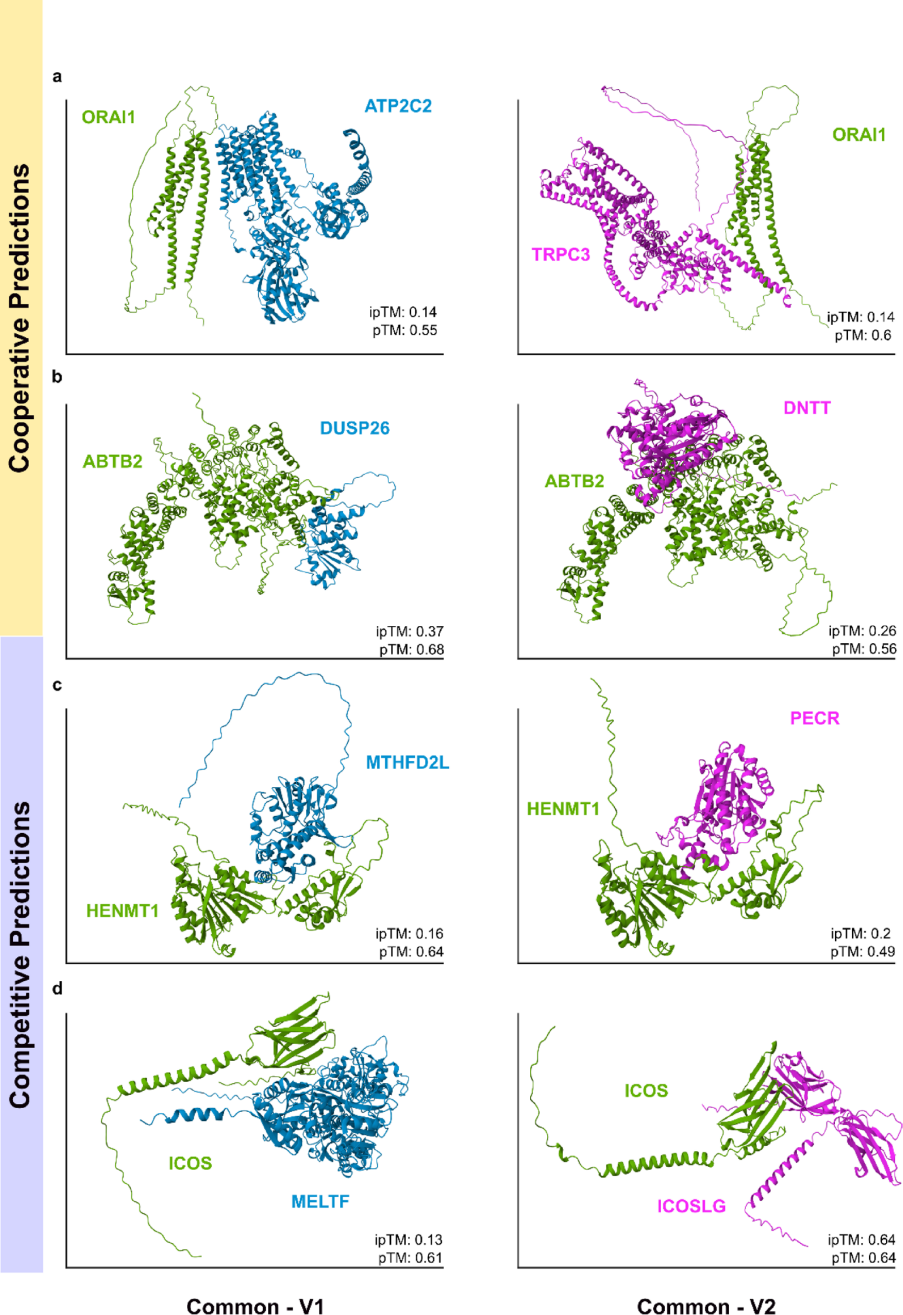
Structural validation of model predictions using AlphaFold. Representative examples of predicted cooperative (top two rows) and competitive (bottom two rows) triplets. Each panel shows a binary complex between the common protein and one of its partners, modeled with AlphaFold 3. In cooperative cases, the two partners bind to distinct, non-overlapping regions on the common protein: (**a**) O75185 (ATP2C2) and Q13507 (TRPC3) binding to Q96D31 (ORAI1); (**b**) Q9BV47 (DUSP26) and P04053 (DNTT) binding to Q8N961 (ABTB2). In competitive cases, both partners share overlapping or adjacent binding interfaces with the common protein: (**c**) Q9H903 (MTHFD2L) and Q9BY49 (PECR) binding to Q5T8I9 (HENMT1); (**d**) P08582 (MELTF) and O75144 (ICOSLG) binding to Q9Y6W8 (ICOS).

To evaluate whether our predictions are meaningful from a structural point of view, we selected four representative triplets for structural modeling: two from the top-ranked cooperative predictions and two from the bottom-ranked competitive predictions, based on their respective classification scores. For each triplet, we generated AlphaFold 3 models of the two binary interactions (common–V1 and common–V2) yielding eight predicted complexes in total (Fig. 4).

We report both pTM and ipTM values as measures of model confidence. While pTM values reflect the confidence in the overall structural fold of the predicted complex, ipTM values focus specifically on the accuracy of the predicted interaction interface, making them particularly informative for our analysis. To further assess the nature of the interfaces, we manually reviewed the predicted structures in PyMOL, examining the positioning of residues at the binding interface. Triplets with non-overlapping binding sites on the common protein were interpreted as cooperative, whereas overlapping or adjacent binding surfaces indicated potential competition between V1 and V2.

The triplet composed of Q96D31 (*ORAI1*) binding to O75185 (*ATP2C2*) and Q13507 (*TRPC3*) was predicted by our model to be cooperative, based on a high classification score, together with favorable pTM and ipTM values for both binary complexes (Fig. 4a). More specifically, ATP2C2 interacts primarily with transmembrane helices of ORAI1 (ipTM = 0.14; pTM = 0.55), while TRPC3 binds to an extracellular region (ipTM = 0.14; pTM = 0.60), with no interface overlap between the two partners. More specifically, ATP2C2 interacts primarily with the cytoplasmic domains of ORAI1, while TRPC3 binds to an extracellular region, with no interface overlap between the two partners. Such an observed spatial configuration aligns with cooperative interactions. ORAI1 is the pore-forming subunit of CRAC channels, central to store-operated calcium entry (SOCE) in immune cells. Its C-terminal domain plays a critical role in STIM1-mediated gating of the channel, and mutations in ORAI1 can cause severe combined immunodeficiency^37,38^. ATP2C2 is an ATPase localized to the Golgi apparatus, where it maintains calcium and manganese homeostasis in the secretory pathway^39^. Although a direct interaction between ATP2C2 and ORAI1 has not previously been described, their co-localization in calcium-regulating compartments suggests a possible functional relationship. TRPC3 is a diacylglycerol-sensitive, non-selective cation channel activated downstream of G protein–coupled receptors (GPCRs) and receptor tyrosine kinases (RTKs)^40^. TRPC3 participates in receptor-operated calcium entry and has been reported to form complexes with ORAI1 and STIM1, integrating different calcium influx pathways^41^. Taken together, the non-overlapping interaction interfaces of ATP2C2 and TRPC3 on ORAI1, combined with their distinct but complementary roles in calcium signaling, support a cooperative mode of interaction. ORAI1 may link multiple calcium signaling pathways through interactions with ATP2C2 and TRPC3 at separate binding sites.

A similar pattern was observed in the second cooperative example of Q8N961 (*ABTB2*) binding to Q9BV47 (*DUSP26*) and P04053 (*DNTT*), where the two partners were positioned at distinct, non-overlapping sites on the common interactor, as revealed by AlphaFold modeling. DUSP26 engaged one end of ABTB2 (ipTM = 0.37, pTM = 0.68), whereas DNTT bound at a different, non-overlapping site (ipTM = 0.26, pTM = 0.56), with clear spatial separation between the binding sites, supporting a cooperative mode of interaction. ABTB2 (Ankyrin repeat and BTB/POZ domain-containing protein 2) contains ankyrin repeat and BTB/POZ domains, which are structural motifs that mediate protein–protein interactions. While direct studies of ABTB2’s interaction partners remain limited, genetic evidence links it to reduced apoptosis in cancer cells^42^. This finding points to a possible role for ABTB2 in promoting cell survival under stress conditions. DUSP26 inactivates MAPK1 (*ERK2*) and MAPK3 (*ERK1*), thereby modulating downstream MAP kinase signaling events. It interacts with the heat shock transcription factor Hsf4b, and through this association, places Hsf4b within a regulatory circuit of the MAP kinase pathway, influencing transcriptional responses to cellular stress^43^. DNTT is a template-independent DNA polymerase that adds non-templated nucleotides during V(D)J recombination, a crucial process for generating diversity in adaptive immune receptors^44^. This analysis indicates that ABTB2 can bind both partners simultaneously, potentially allowing parallel regulation of MAP kinase mediated stress responses and V(D)J recombination (Fig. 4b).

In contrast, two additional triplets classified by the model as competitive provide further structural and biological support for mutually exclusive interactions. The first competitive triplet consists of Q5T8I9 (*HENMT1*) interacting with Q9H903 (*MTHFD2L*) and Q9BY49 (*PECR*) (Fig. 3c). HENMT1 is an RNA methyltransferase that catalyzes the 2′-O-methylation of the 3′ terminal nucleotide of PIWI-interacting RNAs (piRNAs), a modification that enhances their stability and protects them from exonucleolytic degradation^45^. MTHFD2L is a mitochondrial enzyme with dual redox cofactor specificity that functions as a methylenetetrahydrofolate dehydrogenase and methenyltetrahydrofolate cyclohydrolase, participating in the mitochondrial one-carbon metabolism pathway. It is expressed in both adult and embryonic tissues, contributing to nucleotide biosynthesis and cellular metabolic homeostasis^46^. PECR is a peroxisomal enzyme that catalyzes the final step in the NADPH-dependent reduction of trans-2-enoyl-CoAs during fatty acid chain elongation, contributing to lipid metabolism and peroxisomal function^47^. AlphaFold 3 structural modeling revealed that MTHFD2L and PECR bind to overlapping or closely adjacent regions on the surface of HENMT1. Interface analysis showed an ipTM of 0.21 (pTM = 0.61) for the HENMT1–MTHFD2L complex and an ipTM of 0.19 (pTM = 0.58) for HENMT1–PECR, with significant spatial overlap between the binding footprints. This suggests that concurrent binding is prevented by overlap of the interaction interfaces. Given the distinct biological functions of MTHFD2L (mitochondrial one carbon metabolism) and PECR (peroxisomal fatty acid metabolism), such competition may limit HENMT1’s capacity to simultaneously regulate both metabolic pathways.

In the second competitive triplet, Q9Y6W8 (*ICOS*) binds to P08582 (*MELTF*) and O75144 (*ICOSLG*) (Fig. 4d). ICOS is a T-cell co-stimulatory receptor that plays a non-redundant role in adaptive immunity, particularly in T-cell activation and differentiation^48^. Its established ligand, ICOSLG, modulates T-cell activation and trafficking in the tumor microenvironment, contributing to cancer progression and immune regulation^49^. MELTF is a GPI-anchored transferrin-family protein involved in iron binding and transport at the cell surface. Recent studies suggest that MELTF expression can attenuate malignant melanoma progression in both murine models and human patients^50^. AlphaFold 3 modeling indicated that ICOS–ICOSLG forms a higher-confidence interface (ipTM = 0.64; pTM = 0.64) on the ICOS Ig-like head, consistent with its known physiological interaction, which has been partially structurally characterized^51^. By contrast, the ICOS–MELTF interface had much lower prediction confidence (ipTM = 0.13; pTM = 0.61). Structural mapping places MELTF’s binding footprint on the same binding site of the ICOS Ig-domain used by ICOSLG, producing overlapping interfaces, indicating that the two cannot bind simultaneously. Given the established role of ICOSLG as the physiological ligand of ICOS and the distinct functions of MELTF, the observed structural overlap and the higher-confidence ICOS–ICOSLG interface indicate that the two ligands compete for the same binding site, with ICOS.

While our model generally performs well in distinguishing cooperative from competitive triplets, there are cases where caution is warranted. For example, the triplet with P78508 (*KCNJ10*) binding to Q15049 (*MLC1*) and Q96SZ5 (*ADO*) was predicted as competitive based on the classification score. However, AlphaFold 3 structural modeling suggests that MLC1 and ADO bind to different regions on the surface of KCNJ10, suggesting potential cooperation rather than competition engagement (model not shown). This observation shows that even when the model assigns a cooperative or competitive label based on a prediction score, structural modeling can reveal overlapping interfaces that are not captured by network features alone and highlights the importance of structural validation.

## Conclusions

In this study, we systematically explored cooperative and competitive interactions among protein triplets by combining structural data, network topology, hyperbolic embeddings, and machine learning-based prediction. Our results provide novel insights into the structural and functional determinants that differentiate cooperative from competitive assemblies in the human protein interaction network (hPIN). By embedding the hPIN into hyperbolic space, we were able to capture latent geometric properties of the network that traditional biological or topological features alone could not fully reveal. The angular separation between proteins emerged as the most informative feature for predicting interaction type, highlighting the value of hyperbolic geometry in modeling functional organization within complex biological networks. This finding is consistent with previous work showing that angular proximity in hyperbolic space reflects functional similarity, while radial positioning captures evolutionary and topological hierarchy.

Our machine learning models, particularly the Random Forest classifier, demonstrated strong performance in distinguishing cooperative from competitive triplets, achieving high accuracy, F1-score, and AUC values. We observed that biological context such as subcellular localization and the presence of intrinsically disordered regions did not contribute to predictive success as much as geometrical and topological features. Cooperative triplets were enriched in paralogous partners, reflecting their ability to associate with the common protein through non-overlapping interfaces. Importantly, evaluation on an external dataset of newly released structurally supported triplets confirmed the performance of the model and its ability to generalize to previously unseen data.

Validation through AlphaFold 3 demonstrated that cooperative interactions involve distinct interfaces, while competitive interactions are characterized by significant interface overlap. The identification of cooperative modules involved in processes such as ion transport, immune response and cellular stress, strengthens the biological significance of our results. Conversely, competitive triplets showcase the complexity of competition-driven regulatory mechanisms in metabolic pathways and T-cell activation.

A limitation of our approach is the assumption that open triplets not supported by structural complexes represent competitive interactions. While this enabled large-scale modeling, it may introduce false negatives if some of these triplets are cooperative but lack structural evidence. A more accurate strategy would involve identifying cases where two proteins bind overlapping interfaces of a common partner across different PDB entries. However, assembling such a dataset would require extensive structural alignment and is not currently manageable across the whole human interactome. As an alternative, filtering triplets based on the number of L3 pathways connecting V1 and V2 may help improving the training dataset. These refinements represent promising directions for future work.

In conclusion, our study demonstrates that integrating network-based features, hyperbolic embeddings, and machine learning provides a powerful framework for investigating higher-order protein interactions. Our findings demonstrate the potential of geometric approaches as powerful tools for exploring the complexity of biological networks.

## Materials and methods

### Human protein interaction network construction

The human protein–protein interaction network (hPIN) used in this study was built using version 2.3 of the Human Integrated Protein–Protein Interaction rEference (HIPPIE) database^52,53^. HIPPIE integrates experimentally supported physical interactions from major expert-curated resources and assigns a confidence score to each interaction based on evidence type, reproducibility, and publication quality. In this study, the hPIN was constructed using interactions with confidence score ≥ 0.71 because the majority of the edges are supported by more than one experiment^18,24^. Self-interactions were removed, and only proteins within the largest connected component (LCC) were kept. The final network comprises 15,319 proteins and 185,791 high-confidence interactions (Supplementary Tables S1 and S2).

### Mapping the hPIN into the hyperbolic space

We embedded the hPIN in the two-dimensional hyperbolic plane using the R package “NetHypGeom”, which implements the LaBNE + HM algorithm^54^. The algorithm combines manifold learning and maximum likelihood estimation to uncover the hidden geometry of complex networks^55,56^. LaBNE + HM expects a connected network as input, typically the LCC. The other components cannot be mapped due to the lack of adjacency information relative to the LCC. The Popularity–Similarity (PS) model describes the growth of complex networks in hyperbolic space, where radial coordinates reflect node popularity or evolutionary age, and angular coordinates capture functional similarity. In this model, new nodes preferentially connect to existing nodes that are hyperbolically closest to them^20,57^. The network was embedded in H2 to infer the hyperbolic coordinates of each protein, with parameters γ = 2.97, T = 0.83, and w = 2π. The 15,319 nodes of the hPIN lie within a hyperbolic disc where the radial coordinate of a node, ri, represents the popularity dimension with nodes that joined the system first being close to the disc’s center. The angular coordinate, θi, represents the similarity dimension^22^.

### Identification of cooperative interactions and structural annotations

We then identified all the possible open triangles within the hPIN, resulting in approximately 17 million unique triplets. An open triangle is defined as a triplet of proteins in which a central node (common interactor) interacts with two others (V1 and V2), which do not interact. We focused exclusively on open triangles because in closed triangles (where all three proteins interact), no central or common node can be uniquely defined as all nodes are equivalent. This simplified structure allowed a more effective way of modeling cooperative and competitive interaction types.

To annotate structural support for these triplets, we used PPI data from the Interactome3D database, which includes residue-level annotations of experimentally resolved structures deposited in the Protein Data Bank (PDB)^27^. Model-based predictions and self-interactions were excluded from the analysis. Protein complexes were grouped by PDB ID, and only those involving at least three proteins were considered. Within each complex, all possible protein triplets were generated, and the number of observed pairwise interactions per triplet was counted. Triplets in which exactly two of the three possible pairwise interactions were present were classified as cooperative interactions in protein triplet complexes. We then integrated the Interactome3D-derived cooperative triplets with the full set of hPIN open triangles to determine the final set of positive cases used for model training and evaluation. To avoid overrepresentation of proteins, present in many complexes or in complexes with many subunits, only one validated triplet per common interactor was retained, by keeping the first occurrence based on dataset order (Supplementary Table S3). All remaining open triangles in the hPIN were considered non-cooperative (or competitive).

### Feature extraction and randomization

Each protein triplet in the dataset was annotated with a total of 42 features, capturing both network topology and biological context at the node and edge levels. For every protein within a triplet (common interactor, V1, and V2) we extracted 11 features, resulting in 33 features per triplet. These included the two hyperbolic coordinates (r and theta) and four network centrality measures: Degree Centrality (DC)^58^, Closeness Centrality (CC)^59^, Betweenness Centrality (BC)^60^, and Eigenvector Centrality (EC)^61^. These centralities quantify different aspects of a node’s importance within the network, such as how connected it is (DC), how close it is to all other nodes (CC), how often it lies on shortest paths (BC), and how influential its neighbors are (EC). To incorporate biological relevance, we further assigned each protein five additional features: the presence of Intrinsically Disordered Regions (IDRs), obtained from the DisProt database v9.7^62^, and the subcellular localization (nucleus, cytoplasm, endomembrane, and multi-localized) retrieved from the Human Protein Atlas database v24.0^63^.

In addition, three more features were computed for each of the edges between the possible protein pairs in the triplet (common–V1, common–V2, V1–V2): hyperbolic distance (hd), radial difference (rd), and angular difference (thetad), resulting in 9 edge-level features per triplet. To avoid positional bias during model training, the order of V1 and V2 was swapped in a random selection of 50% of the triplets. This ensured that the model learned from meaningful topological and biological properties rather than any artificial ordering patterns. The complete list of features and their descriptions is provided in Supplementary Tables S1 and S2. To access the complete dataset that was used for the modeling see Data Availability section.

### Model development and evaluation

Model development was performed using the caret package in R^64^. The primary classification algorithm applied was Random Forest (RF)^65^, selected for its robustness and effectiveness in handling high-dimensional biological data. To benchmark performance, we additionally trained and evaluated four other machine learning algorithms: Support Vector Machine (SVM)^66^, Logistic Regression (LogReg)^67^, Decision Tree (DT)^68^, and k-Nearest Neighbors (kNN)^69^. The dataset was split into a training set (70%) and a test set (30%), and class imbalance was addressed by applying random undersampling of the majority class during training. Model training was conducted using fivefold cross-validation repeated 10 times to ensure robust hyperparameter optimization. For the Random Forest model, parameters such as the number of variables randomly sampled at each split (n = 22) and the number of decision trees (n = 500) were tuned based on cross-validation performance. Model evaluation was carried out on the independent test set using multiple performance metrics, such as Receiver Operating Characteristic (ROC) curves^70^ and the Area Under the Curve (AUC)^71^ to assess the model’s ability to distinguish between cooperative and competitive interactions. In addition, we calculated accuracy, precision, recall (sensitivity), and F1-score to comprehensively evaluate classification performance, particularly in the context of class imbalance^72^. Finally, we computed feature importance scores using the Random Forest model to identify the most informative topological and biological features contributing to the classification task.

### Homology annotations and external evaluation for biological validation of predicted cooperative and competitive protein triplet complexes

Following the model application to the complete dataset of open triangles in the hPIN, we annotated the predicted interactions with additional biological features to support downstream analysis. Specifically, we integrated information about the paralogy between V1 and V2 proteins and protein sequence lengths. Paralogy between V1 and V2 was assessed using curated human data (GRCh38.p14) from the Ensembl database release 113^73^. In addition, to test the predictive score of the model, we evaluated predictions on an external dataset of structurally supported triplets obtained from the most recent Interactome3D release^27^. Triplets were extracted from newly available complexes following the same criteria as for the training data, and any overlapping cases with the training set were excluded. This dataset provided an independent benchmark of previously unseen triplets for model validation.

### Structural validation of predicted cooperative and competitive protein triplet complexes using AlphaFold 3

Following the prediction of protein triplet complexes as either cooperative or competitive, we proceeded with structural modeling to evaluate whether the predicted interaction types were supported by structural evidence. For each protein triplet, the individual protein sequences were retrieved from the UniProtKB database^74^ and used as input for AlphaFold 3^75^. Instead of modeling the entire triplet as a single complex, we performed pairwise structure prediction by separately analyzing the common-V1 and common-V2 protein pairs. Specifically, we examined whether V1 and V2 interacted with the same or overlapping regions on the common interactor protein. Overlap in binding sites was interpreted as evidence of potential competitive binding, while distinct binding interfaces were indicative of potential cooperative interactions. We used PyMOL to visualize the predicted structures, which allowed us to manually examine where the proteins were binding and how they were positioned in 3D space^76^.

## Supplementary Information

Below is the link to the electronic supplementary material.

Supplementary Material 1

Supplementary Material 2

## Data Availability

All supplementary data supporting the findings of this study, including node- and edge-level features, structural annotations, and prediction scores, are available at: https://github.com/avagiona/Cooperative-Competitive-interactions-within-protein-triplets.
