# Supplementary material for: Unraveling cooperative and competitive interactions within protein triplets in the human interactome
Source: Sci Rep. 2025 Sep 15;15:32548. doi: 10.1038/s41598-025-19264-4 (PMC12436631; doi:10.1038/s41598-025-19264-4)
Supplement: Supplementary file 1 — Supplementary Material 1 [file 41598_2025_19264_MOESM1_ESM.pdf]

Supplementary Materials for manuscript:

Unraveling cooperative and competitive interactions within protein triplets in the human interactome

Aimilia-Christina Vagiona, Pablo Mier, Miguel A. Andrade-Navarro

SUPPLEMENTARY TABLE LEGENDS

Supplementary Table S1. Nodes of the hPIN. Columns indicate protein identifiers (UniProtKB), hyperbolic coordinates (r, theta), centrality measures (Degree Centrality—DC, Closeness Centrality—CC, Betweenness Centrality—BC, and Eigenvector Centrality—EC), intrinsic disorder region presence (idr; yes=1, no=0) and subcellular localization (nucleus, cytoplasm, endomembrane, multi-localized proteins).

Supplementary Table S2. Edges of the hPIN. Columns indicate protein identifiers (UniProtKB; V1, V2), hyperbolic distance (hd), r difference (rd) and angular difference (thetad) derived from the hyperbolic embedding.

Supplementary Table S3. Structurally supported protein triplets annotated using Interactome3D. Columns indicate the triplet identifier (Triplet_ID), associated PDB structure (PDB_ID), the common protein and its interactors (Common, V1, V2), and the regions of interaction between the common protein and each interactor (region_common_V1, region_common_V2), specifying the PDB chain and residues involved.

Supplementary Table S4. Predictions of cooperative and non-cooperative protein triplets with associated scores. Columns indicate whether structural evidence supports the interaction (cooperative: 1 for structurally validated, 0 otherwise), the protein identifiers (common, V1, V2), and the classification scores assigned by the predictive model for each class (score_competitive and score_cooperative).

Supplementary Table S5. Predictions of cooperative and competitive protein triplets based on low-degree proteins (DC≤10) triplets with structural validation and feature annotations. Columns include the validation label based on structural evidence (cooperative: 1 for structurally validated, 0 otherwise), protein identifiers (common, V1, V2), angular difference between V1 and V2 in hyperbolic space (thetad_V1_V2), degree centralities of each protein (degree_common, degree_V1, degree_V2), prediction scores (score_competitive and score_cooperative), quantile (quantile) and presence of paralogous relationships (paralog).
